# Multifunctional MXene/C Aerogels for Enhanced Microwave Absorption and Thermal Insulation

**DOI:** 10.1007/s40820-023-01158-7

**Published:** 2023-08-09

**Authors:** Fushuo Wu, Peiying Hu, Feiyue Hu, Zhihua Tian, Jingwen Tang, Peigen Zhang, Long Pan, Michel W. Barsoum, Longzhu Cai, ZhengMing Sun

**Affiliations:** 1 School of Materials Science and Engineering, Southeast University, Nanjing, 211189 People’s Republic of China; 2 Department of Materials Science & Engineering, Drexel University, Philadelphia, PA 19104 USA; 3 The State Key Laboratory of Millimeter Waves, School of Information Science and Engineering, Southeast University, Nanjing, 210096 People’s Republic of China

**Keywords:** MXene, Microwave absorption, Aerogel, Radar cross-sectional (RCS) simulation, Thermal insulation

## Abstract

Two-dimensional transition metal carbides and nitrides (MXene) have emerged as promising candidates for microwave absorption (MA) materials. However, they also have some drawbacks, such as poor impedance matching, high self-stacking tendency, and high density. To tackle these challenges, MXene nanosheets were incorporated into polyacrylonitrile (PAN) nanofibers and subsequently assembled into a three-dimensional (3D) network structure through PAN carbonization, yielding MXene/C aerogels. The 3D network effectively extends the path of microcurrent transmission, leading to enhanced conductive loss of electromagnetic (EM) waves. Moreover, the aerogel’s rich pore structure significantly improves the impedance matching while effectively reducing the density of the MXene-based absorbers. EM parameter analysis shows that the MXene/C aerogels exhibit a minimum reflection loss (RL_min_) value of − 53.02 dB (*f* = 4.44 GHz, *t* = 3.8 mm), and an effective absorption bandwidth (EAB) of 5.3 GHz (*t* = 2.4 mm, 7.44–12.72 GHz). Radar cross-sectional (RCS) simulations were employed to assess the radar stealth effect of the aerogels, revealing that the maximum RCS reduction value of the perfect electric conductor covered by the MXene/C aerogel reaches 12.02 dB m^2^. In addition to the MA performance, the MXene/C aerogel also demonstrates good thermal insulation performance, and a 5-mm-thick aerogel can generate a temperature gradient of over 30 °C at 82 °C. This study provides a feasible design approach for creating lightweight, efficient, and multifunctional MXene-based MA materials.

**Supplementary Information:**

The online version contains supplementary material available at 10.1007/s40820-023-01158-7.

## Highlights

- Curving 2D MXene into 1D nanofibers can effectively stop the restacking of MXene flakes, and then the nanofibers are used to construct a lightweight and multifunctional MXene/C aerogel.
- The MXene/C aerogels achieved an RL_min_ of − 53.02 dB and EAB of 5.3 GHz. The radar cross-sectional reduction value of MXene/C aerogels can reach 12.02 dB m^2^.
- Integrating multiple functions such as thermal insulation, sensing, and microwave absorption into one material—MXene/C aerogel.

**Supplementary Information:**

The online version contains supplementary material available at 10.1007/s40820-023-01158-7.

## Graphical abstract

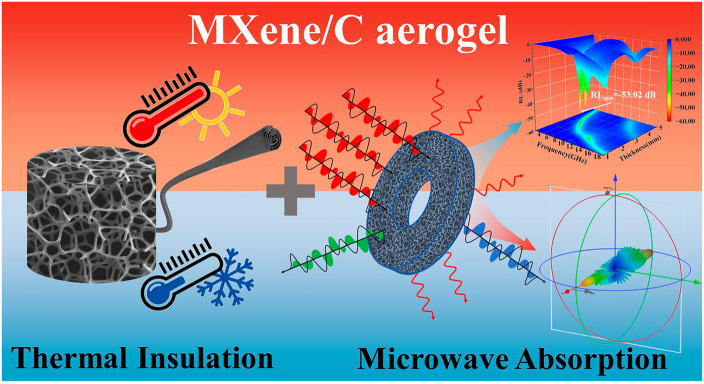

## Introduction

The expansion of wireless communication technology has driven significant social progress, and the emergence of new communication technologies, such as 5G, has brought unprecedented convenience to human life [1–3]. However, it has also exacerbated the issue of electromagnetic (EM) pollution, which can disrupt the normal functioning of electronic devices and pose a potential threat to human health [2, 4, 5]. To cope with this problem, it is crucial to develop efficient microwave absorption (MA) materials [6–10], which can transform EM waves into heat and subsequently dissipate them [10]. MA materials are widely used in military, aviation, communication, electronics, medicine, and other fields, and have been developing toward the direction of “thin, light, wide and strong” [11–15]. To meet the demands of emerging electronics, MA materials are being required to possess other functionalities, such as thermal insulation, sensing capability and others [16, 17].

The rapid development of novel materials including magnetic metals/alloys and ferrites [18], carbon-based materials [19], metal–organic frameworks (MOFs) [3, 20], and two-dimensional (2D) transition metal carbides/nitride (MXene) [21] has significantly advanced the field of MA technology. Among them, MXene has emerged as a highly promising MA material due to its exceptional features, such as excellent conductivity, unique two-dimensional structure, large specific surface area, and abundant functional groups [5, 21–23]. In a pioneering study by Qing [24], 50 wt% Ti_3_C_2_T_x_/epoxy composite material with a thickness of 1.4 mm exhibited a minimum reflection loss (RL_min_) of − 11 dB within the range of 12.4–18 GHz, highlighting the high MA performance of MXene. However, the MA capability of MXene remains restricted by the issue of impedance mismatch caused by its high conductivity [25, 26]. The impedance matching and EM waves attenuation ability are the two critical factors that determine the MA performance [27]. Impedance matching ensures that EM waves can enter the absorber, which is prerequisite for the subsequent losses. The excellent electrical conductivity of MXene can enhance its conductive losses to EM waves, but it also can lead to impedance mismatching [28]. Additionally, the strong van der Waals interaction between MXene nanosheets causes them tend to self-stacking, which hinders the multiple reflections and scattering of EM waves, ultimately resulting in a significant reduction in the MA capacity of MXene.

Constructing three-dimensional (3D) network structures is regarded as an essential strategy for resolving the issue of impedance mismatch, while also helping to create lightweight MA materials. According to the Maxwell–Garnett theory [29], 3D network materials can be considered as composite materials consisting of solid and air. Increasing the volume of air can lower the material’s dielectric constant, which can improve the impedance matching [30–33]. Therefore, a series of 3D-structured MXene-based MA materials were synthesized through template method [34], self-assembly [35], spray drying [36], and other methods [32]. Wang et al. [37] prepared graphene/Ti_3_C_2_T_x_ MXene hybrid aerogels with a 3D cross-linked porous structure using a combination of hydrothermal and freeze-drying methods. The unique aerogel structure significantly improved the 3D network conductivity, dipole and interface polarization, and multiple scattering, resulting in an RL_min_ value of − 31.2 dB at 8.2 GHz and an effective absorption bandwidth (EAB) of 5.4 GHz at only 2.05 mm thickness. Similarly, Li et al. [38] fabricated mixed aerogel microspheres composed of graphene oxide and Ti_3_C_2_T_x_ MXene through fast-freezing-assisted electrospinning. The aerogel structure not only made the absorber lightweight but also prolonged the attenuation path when EM waves were injected, resulting in an RL_min_ of − 49.1 dB at 14.2 GHz with a lower filling load of 10.0 wt% and a thickness of 1.2 mm. The strategy mentioned above can efficiently produce 3D MXenes structure, but it does not completely solve the issue of MXene nanosheets self-stacking. Furthermore, due to the lack of robust connections, the mechanical stability of the 3D network structure is weak, which significantly hinders the application of MXene-based MA materials. By curling the 2D MXene into a one-dimensional (1D) configuration, we can not only entirely eliminate the stacking of MXene layers but also efficiently establish a 3D interconnected network.

In this work, a facile and controllable electrospinning technique is employed to fabricate MXene/C aerogels with 3D network structure. 2D MXene nanosheets were curled into 1D nanofibers, effectively avoiding self-stacking of MXene nanosheets. The subsequent carbonization of PAN leads to the formation of interlinked 3D conductive networks of the MXene/C nanofibers. The 3D conductive network can extend the current transmission path and enhance conductivity loss; and, the internal porous structure creates abundant heterogeneous interfaces, strengthening interface polarization; the large number of pore wall structures can also promote multiple reflection loss of EM waves. Benefiting from its unique structural advantages and the synergistic effect of its components, the MXene/C aerogel displays remarkable MA performance. Furthermore, the strong binding between MXene/C nanofibers imparts good structural stability to the aerogel, enabling stable MA performance. The excellent thermal insulation and infrared stealth properties of the aerogel align with the multifunctional development requirements of MA materials.

## Experimental Procedure

### Materials and Chemicals

Ti_3_AlC_2_ (325 mesh, 99%) was purchased from Forsman Scientific Co., Ltd, China. Lithium fluoride (LiF, 99%) and Tetrabutylammonium hydroxide (TBAOH, 40% in Water) were from Aladdin Reagent Co. Ltd, China. Hydrochloric acid (HCl, 36–38%) was from Sinopharm Chemical Reagent Co., Ltd, China. Polyacrylonitrile (PAN, Mw = 15,000) and *N*,*N*-dimethylformamide (DMF, 99%) were supplied from Macklin Biochemical Co., Ltd, China.

### Synthesis of Ti_3_C_2_T_x_ MXene Nanosheets

Ti_3_C_2_T_x_ MXene nanosheets were synthesized by selective etching the Al layer from the Ti_3_AlC_2_ powder with LiF/HCl. In detail, 20 mL of hydrochloric acid (9 M) was added to a Teflon vial. Subsequently, 1 g of LiF was slowly added to the vial with magnetic stirring (500 rpm). Then, 1 g of Ti_3_AlC_2_ powder was added to the solution, and the etching reaction lasted for 24 h at 40 °C. When the reaction was completed, the product was collected by centrifugation (4000 rpm, 5 min for each round), and then washed with deionized water until the pH reached 7. The precipitate was then sonicated for 1 h in 100 mL of deionized water under Ar atmosphere. Then, a delaminated Ti_3_C_2_T_x_ (*d*-Ti_3_C_2_T_x_) suspension was collected by removing precipitates.

To effectively disperse Ti_3_C_2_T_x_ in DMF, we employed TBAOH to modify its surface functional groups. This modification involves the replacement of a majority of the –OH surface groups in Ti_3_C_2_T_x_ with TBA^+^ ions, which enhances the hydrophobicity of Ti_3_C_2_T_x_ and improves its dispersibility in DMF [39]. 1 g of *d*-Ti_3_C_2_T_x_ powder was mixed with 24 mL of 25% TBAOH solution and stirred at room temperature for 4 h. Any excess TBAOH was then removed by cleaning the mixture with ethanol, and all the resulting precipitate was collected in a 50 mL centrifuge tube. Finally, the precipitate was vacuum dried at 80 °C for 24 h.

### Preparation of MXene/C Aerogels

**Fig. 1:**
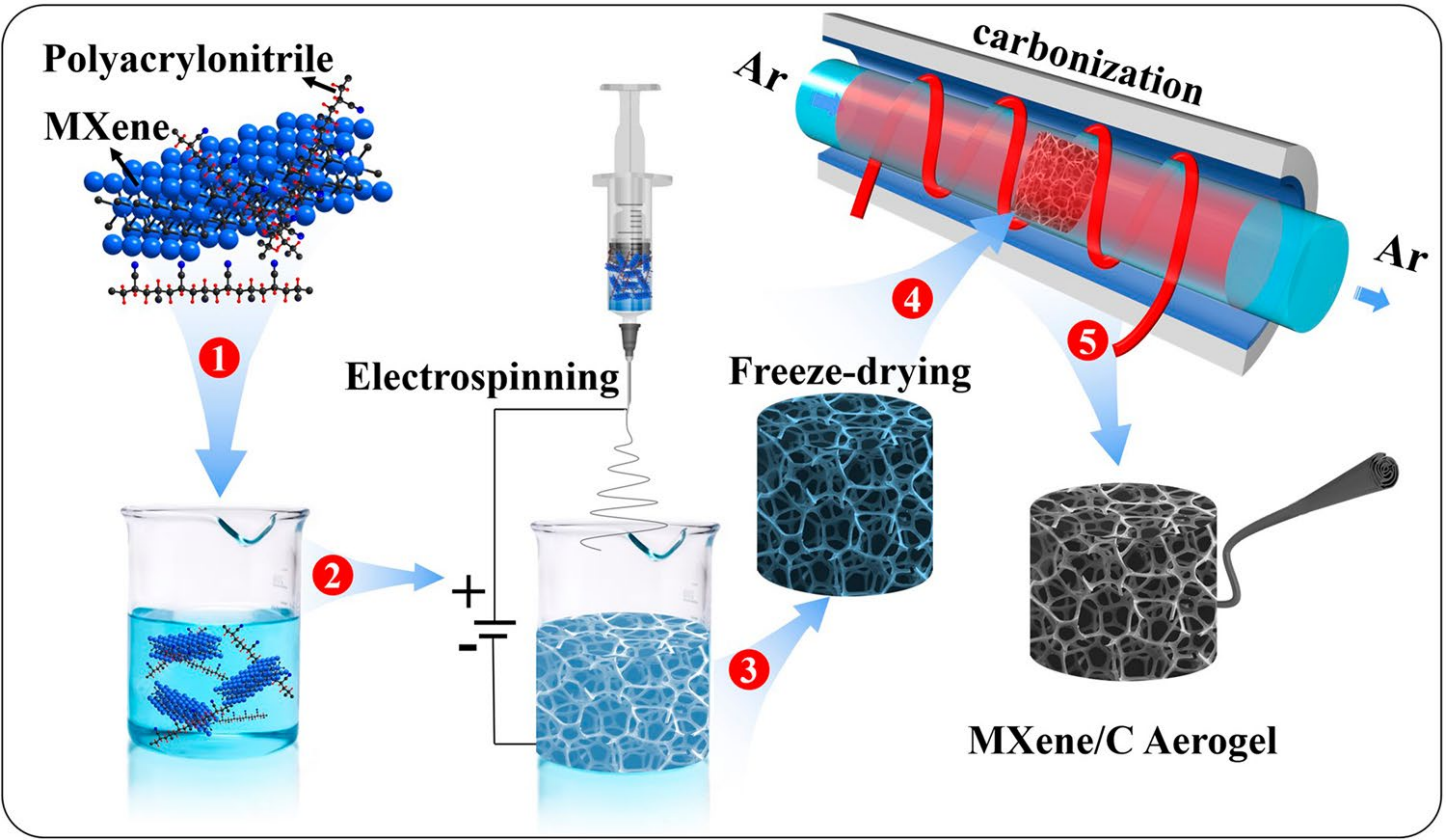
Schematic of the fabrication process of MXene/C aerogels

Figure 1 shows a schematic illustration of the preparation process for the MXene/C aerogels. Firstly, PAN and the surface-modified Ti_3_C_2_T_x_ powder were added to a DMF solution and subjected to magnetic stirring for 24 h to obtain the MXene/PAN/DMF electrospinning solution. Next, the spinning solution was transferred to a plastic syringe with a 21G metal nozzle. A metal collector plate was placed in deionized water in a glass container. A voltage of + 20 kV was applied to the nozzle, and − 0.5 kV was applied to the collector plate. The nozzle was positioned 10 cm vertical to the water surface, and the syringe feeding rate was set at 1 mL/h. The electrospun MXene/PAN nanofibers were transferred to a 50 mL beaker containing deionized water and frozen at − 40 °C for 12 h. Finally, the frozen MXene/PAN nanofibers were then freeze–dried for 72 h to obtain the MXene/PAN aerogels. The aerogels were then placed in to a ceramic boat and subjected to pre-oxidation at 250 °C for 1 h with a heating rate of 2 °C min^−1^ under air atmosphere. Subsequently, they were annealed at 700 °C for 1 h under Ar atmosphere. There aerogels, labeled as MC-1, MC-2, and MC-3, were obtained, corresponding to raw electrospinning solutions with PAN:Ti_3_C_2_T_x_ of 5:1, 2:1, and 1:1 in mass ratio, respectively.

### Materials Characterization

Phase constitution of the aerogels was characterized using X-ray diffractometry (XRD, Haoyuan, DX-2700BH). Their surface composition was characterized using laser Raman spectrometry (Witec Alpha300, *λ* = 532 nm). Their microstructure was characterized using scanning electron microscopy (SEM, FEI Sirion 200) and high-resolution transmission electron microscopy (HRTEM, FEI TALOS F200X). To measure their electromagnetic parameters, a vector network analyzer (ANRITSU MS46322B) was used in the frequency range of 2–18 GHz. A movable square mold was designed and used to compress the sample. First, aerogels of equal mass are compressed into the same volume to ensure that aerogels have the same density. Subsequently, the molten paraffin wax of equal mass into the mold, allowing it to cool and solidify, thus obtaining absorbers with the same filling ratio (15 wt%). These absorbers were then pressed using a mold to form coaxial rings with an outer diameter of 7.00 mm and an inner diameter of 3.04 mm. The reflected loss value (RL) of the material was calculated using the transmission line theory. The formulae used for the calculation are as follows [17]:

1 $${\text{RL}} = 20 \log \left| {(Z_{{{\text{in}}}} - Z_{0} )/(Z_{{{\text{in}}}} + Z_{0} )} \right|$$

2 $$Z_{{{\text{in}}}} = Z_{0} (\mu_{r} /\varepsilon_{r} )^{1/2} \tanh \left[ {j(2\pi fd/c)(\mu_{r} \varepsilon_{r} )^{1/2} } \right]$$

where *Z*_in_ is the input impedance of the absorber surface, *Z*_0_ is the air impedance, *f* is the microwave frequency, *t* is the thickness of the absorber, and *c* is the speed of light in a vacuum. *μ*_*r*_ (*μ*_*r*_=*μʹ*−*jμ″*) and *ε*_*r*_ (*ε*_*r*_=*εʹ*−*jε″*) are complex permittivity and complex permeability, respectively. The pressure sensing performance of the aerogel was tested using an electronic universal testing machine (MTS, EXCEED Model E43) and an electrochemical workstation (GAMRY, INTERFACE 1010 E). Meanwhile, a thermal infrared imaging camera (FLIR, T420) was employed to test the thermal insulation performance of the MXene/C aerogels.

### RCS Simulation

The radar cross-sectional (RCS) of the MXene/C aerogels under real far-field conditions was simulated using CST Studio Suite 2020. The simulation model comprised a double-layered flat plate (20 cm × 20 cm), featuring a 2.0 mm absorbing layer (MXene/C aerogels) on top and a 1.0 mm perfectly electrically conductive (PEC) layer at the bottom. The aerogel/PEC model plate was positioned on the X-O-Y plane, with the plane EM waves being incident along the negative direction of the Z-axis at an angle ranging from − 60° to 60°. Open boundary conditions were set in all directions, with the monitor frequency fixed at 12 GHz. The RCS can be expressed in the following equations [16]:

3 $$\sigma (m^{2} ) = \mathop {\lim }\limits_{R \to \infty } 4\pi R^{2} \left( {\left| {\frac{{E_{s} }}{{E_{i} }}} \right|} \right)^{2} = \mathop {\lim }\limits_{R \to \infty } 4\pi R^{2} \left( {\left| {\frac{{H_{s} }}{{H_{i} }}} \right|} \right)^{2} = \mathop {\lim }\limits_{R \to \infty } 4\pi R^{2} \frac{{S_{s} }}{{S_{i} }}$$

where *E*_s_ and *E*_i_ stand for the intensities of the scattered electric field and incident electric field, respectively. *H*_s_ and *H*_i_ represent the intensities of the scattered magnetic field and incident magnetic field, respectively. *S*_s_ and *S*_i_ are the intensities of the scattered electric field and incident electric field, respectively.

## Results and Discussion

### Characterization of Composition and Microstructures

**Fig. 2:**
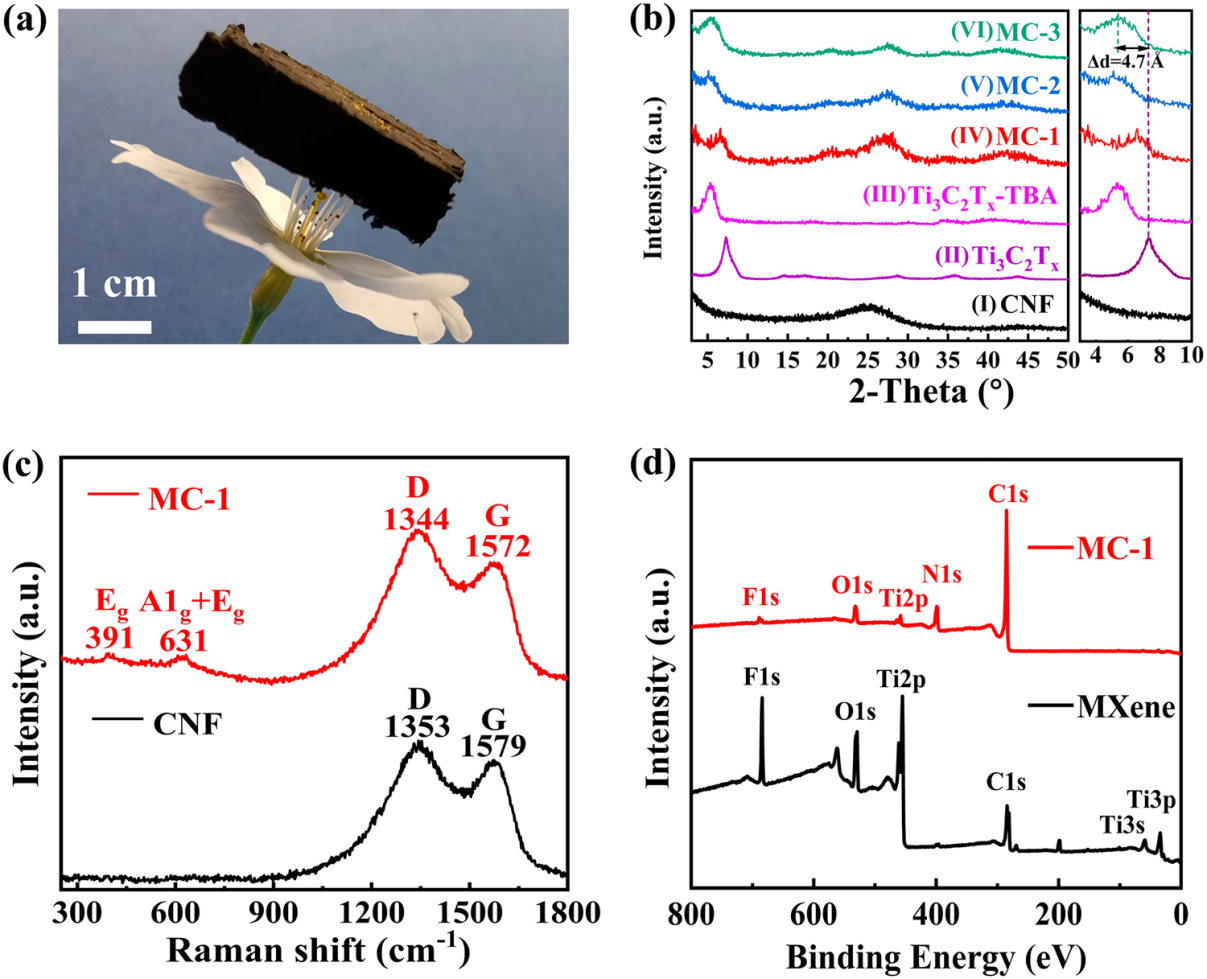
**a** Digital image of a MXene/C aerogel standing on a stamen. **b** XRD patterns of as-prepared samples and magnified image. **c** Raman spectra of CNF and MC-1 aerogel. **d** XPS spectrum of MXene and MC-1aerogel

As depicted in Fig. 2a, the MXene/C aerogels with a calculated density ranging from 0.02 to 0.05 g cm^−3^ can be supported by the stamens of a cherry blossom. The broad peak between 20° and 30° of the XRD pattern of the carbon nanofiber (CNF) (Fig. 2b, I) was attributed to the amorphous carbon formed by the carbonization transformation of PAN [40]; the peak at around 6° (Fig. 2b, II) is indexed to the (002) plane of Ti_3_C_2_T_x_ [21]; the XRD patterns of the MXene/C aerogels (Fig. 2b, IV–VI) showed both the amorphous carbon peak and the (002) plane diffraction  peak of Ti_3_C_2_T_x_ MXene, signifying the successful preparation of the MXene/C binary composite material. As shown in Fig. 2b, the (002) peak of MC-3 exhibits an apparent left shift compared to the bare Ti_3_C_2_T_x_ MXene, indicating an enlargement of its interlayer spacing. Calculations based on the XRD data reveal that the interlayer spacing of Ti_3_C_2_T_x_ increased by 39.1% (from its original value of 12.0 to 16.7 Å). This suggests that this method can effectively eliminate the stacking of 2D MXene. Furthermore, the high-temperature heating process can cause the stacking of MXene samples, whether they are bare or intercalated. Figure S1a shows that after heating the bare Ti_3_C_2_T_x_ and TBAOH-modified Ti_3_C_2_T_x_ at 700 °C, the (002) peak exhibits a significant right shift, indicating a reduction in their interlayer spacing. In comparison, the (002) peak of Ti_3_C_2_T_x_/PAN, prepared through electrospinning, does not exhibit any significant shift before and after carbonization. This observation further supports the evidence that the process of curing 2D MXene nanosheets into 1D nanofibers can effectively eliminate MXene layer stacking. Figure S1b shows the XRD patterns of Ti_3_C_2_T_x_ MXene after pre-oxidation, carburization, which retain the characteristic diffraction peaks of Ti_3_C_2_T_x_ without the presence of TiO_2_ diffraction peaks. This indicates that significant oxidation did not occur in MXene after pre-oxidation and carburization.

Raman spectra were contained in Fig. 2c. The vibration peaks at 1353 and 1579 cm^−1^ for the CNF  corresponded to the D peak of amorphous carbon and the G peak of graphite carbon, respectively [41]. The ratio of the peak value (*I*_D_/*I*_G_) was used to evaluate the degree of graphitization, and the *I*_D_/*I*_G_ value for the CNF was 1.06. The D and G peaks of the MC-1 aerogel were located at 1344 and 1572 cm^−1^, respectively, and its *I*_D_/*I*_G_ value (1.09) was similar to that of CNF. Thus, the incorporation of MXene did not alter the degree of graphitization of the sample. The Raman shift peaks of MC-1 at 391 and 631 cm^−1^ represent the *E*_g_ group vibration, including the in-plane (shear) modes of Ti and C, respectively; the peak at 631 cm^−1^ also corresponded to the A_1g_ symmetric out-of-plane vibration of C atoms [40].

The XPS spectrum (Fig. 2d) of MXene/C reveals that it is primarily composed of C, Ti, O, and F elements, which is consistent with the composition of the MXene. The presence of F elements in MXene/C originates from the F-functional groups on the surface of MXene. Additionally, in the XPS spectrum of MXene/C, there are peaks corresponding to N element. The N element originates from PAN in the spinning solution and is doped into carbon during the carbonization process of PAN, forming point defect [42]. These F-functional groups and point defects can enhance dipole polarization and N defect polarization under high-frequency EM fields, which is beneficial for increasing the material’s MA capacity [35]. Additionally, when comparing the Ti 2*p* XPS spectra of Ti_3_C_2_T_x_ MXene and MC-1 (Fig. S2), it can be observed that the intensity of the TiO_2−x_F_x_ bond slightly increases, indicating a slight oxidation of Ti_3_C_2_T_x_ MXene. However, the composition of MC-1 is still dominated by the formation of unoxidized MXene.

**Fig. 3:**
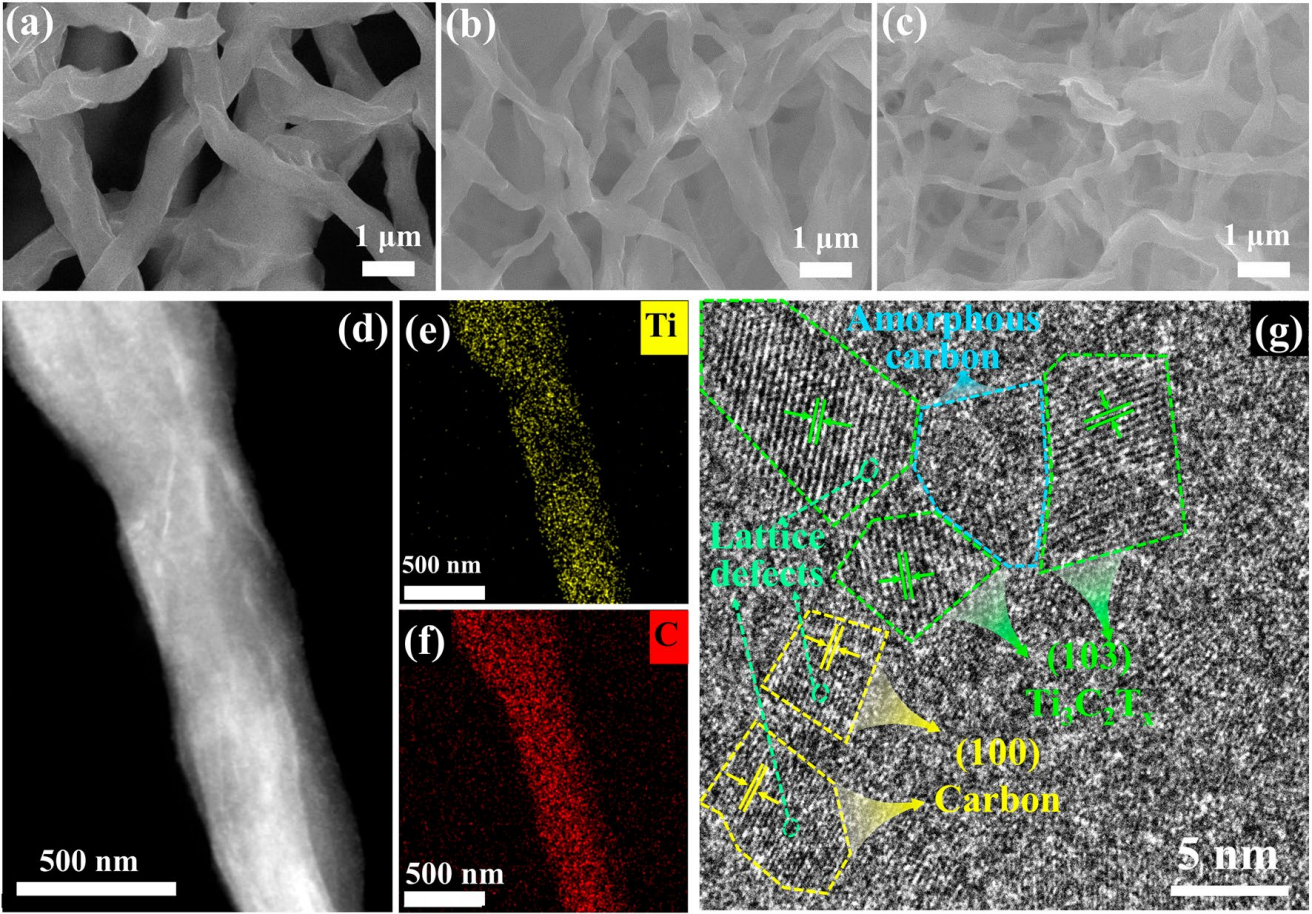
SEM images of **a** MC-1, **b** MC-2 and **c** MC-3 aerogel. **d** TEM image of the MXene/C aerogel, and **e, f** element mapping images. **g** HRTEM image of MC-1 aerogel

SEM images of CNF (Fig. S1a, b) displayed a smooth surface and a diameter ranging from 0.5 to 1.0 μm, while, as shown in Fig. 3a–c, the MXene/C nanofibers have wrinkled surfaces. The MXene nanosheets curled during the spinning process, which resulted in the wrinkles. In addition, the diameter of MXene/C nanofibers decreased as the MXene content increased, with the MC-3 aerogel displaying a minimum diameter of around 100 nm. Moreover, MXene/C nanofibers are interconnected to form a 3D network structure, as shown in Fig. 3a–c, which can limit the movement of the nanofibers, rendering the aerogel exceptional mechanical properties such as elasticity. The high-resolution TEM image of MC-3 aerogel (Fig. 2d) shows the MXene nanosheets constrained inside the fibers. The corresponding element mapping (Fig. 2e, f) provides further evidence that MXene and carbon are the constituents of the aerogels. In the HRTEM image, clear lattice stripes of 0.24 and 0.22 nm can be observed, corresponding to the MXene (103) and carbon (100) planes. Together with the element mapping, this image can further indicate that the main components of the MXene/C nanofiber are Ti_3_C_2_T_x_ MXene and carbon. Additionally, numerous heterointerfaces (crystal/amorphous interfaces) and lattice defects (such as lattice distortion, discontinuous lattice fringes and point defects) are present [43]. These regions exhibit disrupted lattice periodicity, leading to uneven charge distribution and changes in the spatial charge regions [44–47]. As a result, interface polarization and electromagnetic wave loss occur.

### Mechanical Property

**Fig. 4:**
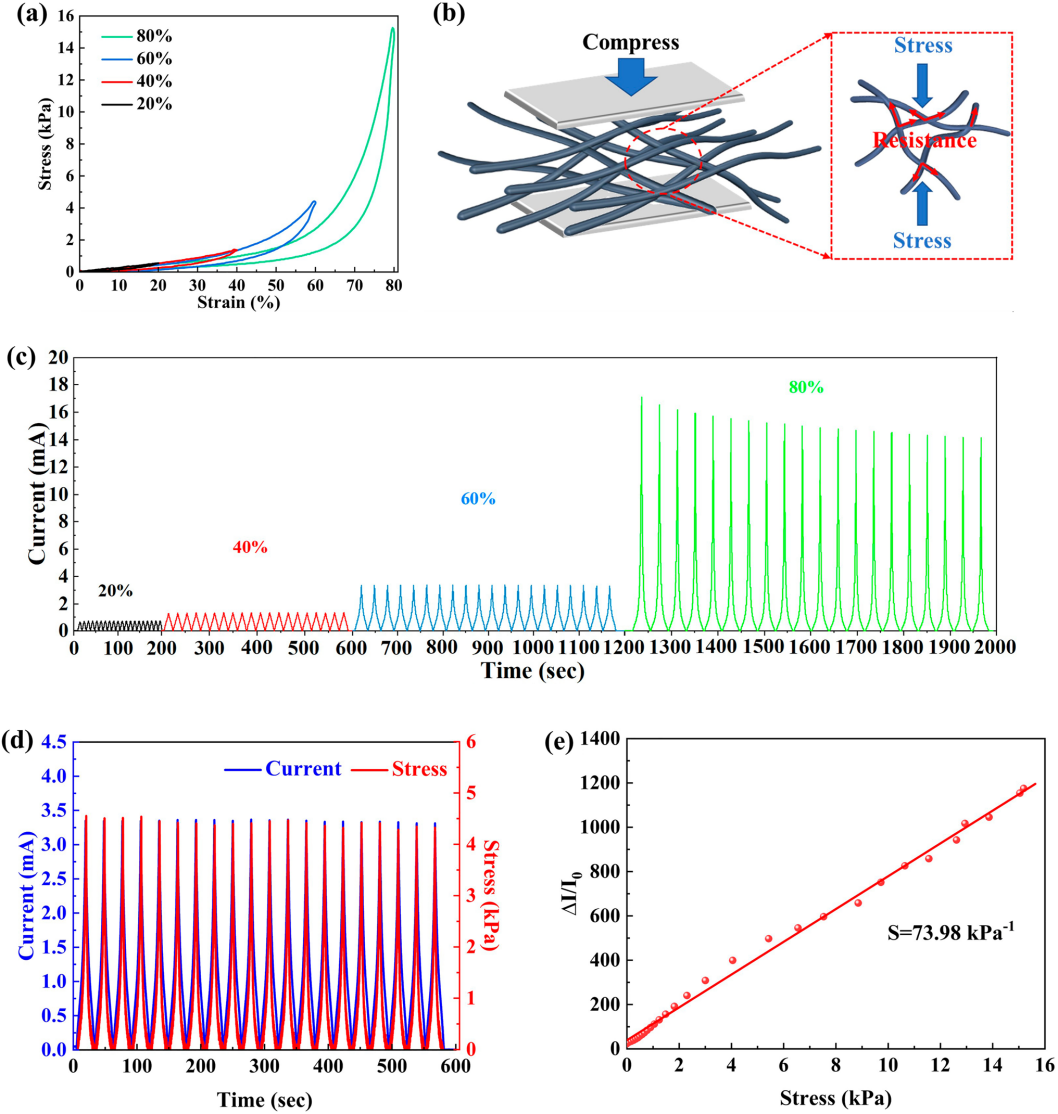
**a** Stress–strain curves. **b** Schematic diagram of the response mechanism of MXene/C nanofibers to external stress. **c** Current response at different strains. **d** Synchronous response of current under stress. **e** Linear sensitivity with a working pressure range of 0–15.19 kPa

Structural stability of MA material is essential for maintaining its MA properties, which requires the material possesses sufficient mechanical performance [48]. Therefore, the mechanical properties of MXene/C aerogels were investigated, as shown in Fig. 4. The MC-2 aerogel can withstand stresses of 0.56, 1.47, 4.41 and 15.19 kPa at 20%, 40%, 60% and 80% compressive strains, respectively. Moreover, Fig. S4 shows that MXene/C aerogels possess remarkable resilience. Even after undergoing significant compression, the aerogel quickly returns to its original state without damage. The characterization results of the microstructure of MXene/C aerogels show that interconnected MXene/C nanofibers form a robust 3D network. The response mechanism of MXene/C nanofibers to external stress is shown in Fig. 4b. The robust crossing points of 3D network can be well retained during the deformation, thus making the aerogel exhibit excellent resilience.

In addition to its excellent resilience, the 3D network also has to well retain its electrical conductivity after undergoing external forces, which is important for its MA performance. When pressed, more fibers within the 3D network come into contact, resulting in a decrease in resistance. When released, the 3D network returns to its original state, thereby restoring resistance. Therefore, by monitoring the changes in the resistance of the aerogel under pressure, changes in the internal structure of the 3D network can be detected. Figure 4c shows the real-time current response of the MC-2 aerogel under 20%, 40%, 60%, and 80% compressive strains, respectively, and the current response curves demonstrate a stable periodic change. In particular, with 20 cycles at 60% compressive strain, the perfect periodic change of the current with stress (Fig. 4d) indicates the ultra-high fatigue resistance performance, further proving its excellent structural stability of the 3D network. The robustness against the large compressive strain and excellent anti-fatigue performance makes the MXene/C aerogel a promising material for pressure sensors. The sensitivity (*S*) is a crucial indicator used to assess the performance of pressure sensors, and it is defined *S* = *δ*(ΔI/I0)/*δp* [49]. As shown in Fig. 4e, the linear sensitivity of MC-2 aerogel can reach 73.98 kPa^−1^ within the pressure range of 0–15.19 kPa, implying that the aerogel can accurately detect current output signals across a wide strain range of 0–80%. As a result of the design of a robust 3D conductive network, MXene/C aerogels exhibit remarkable electrical conductivity and resilience, making them highly suitable for achieving efficient and stable MA performance.

### Microwave Absorption Performance

**Fig. 5:**
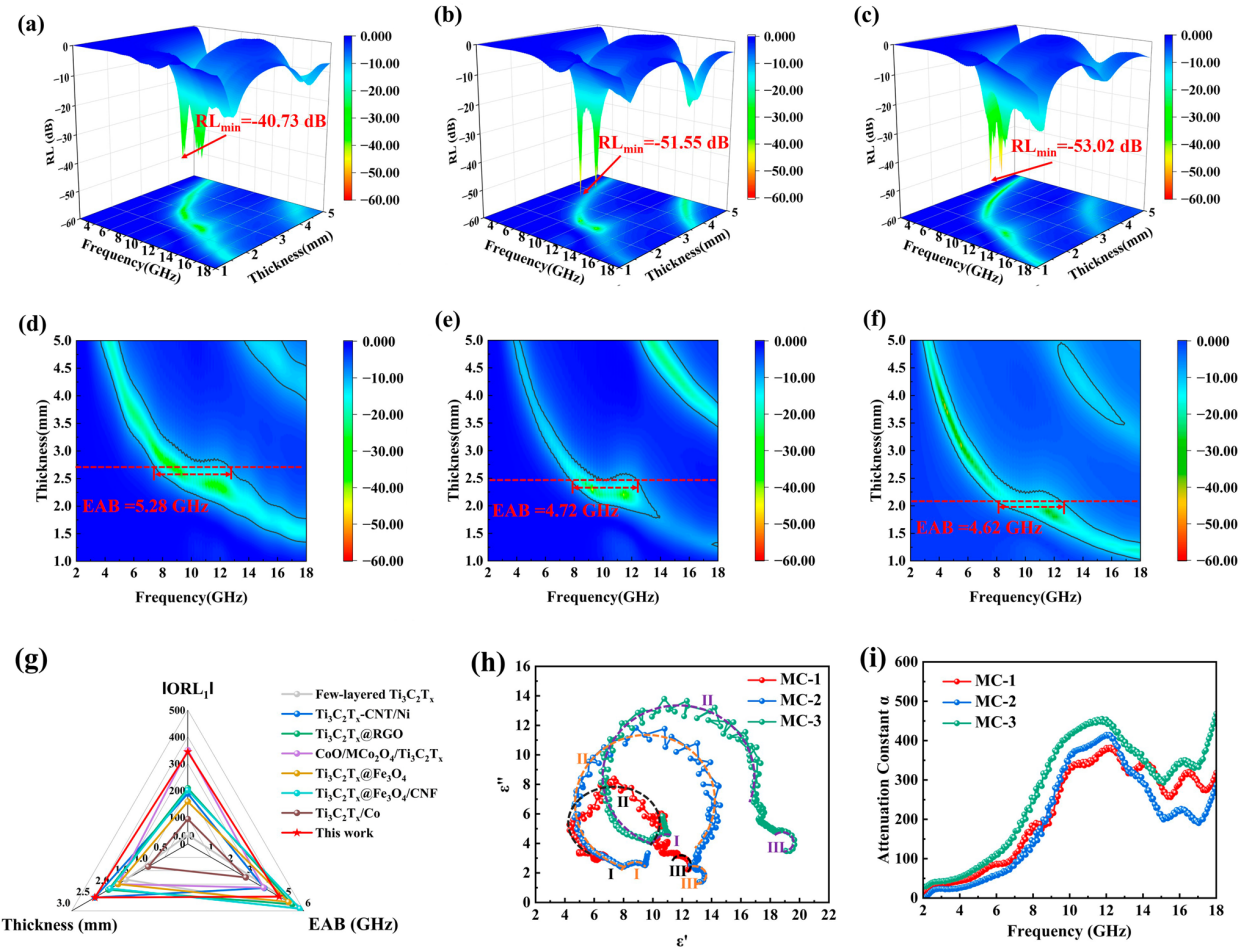
3D representation of RL values of MXene/C aerogels: **a** MC-1, **b** MC-2 and **c** MC-3. 2D representation of RL values of MXene/C aerogels: **d** MC-1, **e** MC-2 and **f** MC-3. **g** Comparison of the MA properties of various related materials [24, 37, 43, 50–53]. **h** Cole–Cole semicircle and **i** Attenuation constant of MXene/C aerogels

The commonly used as assessment metrics for the MA performance of absorbers involves the RL_min _and EAB values. The RL values of CNF, MXene, and MXene/C aerogels were calculated using Eqs. (1 and 2) and presented in Figs. S5a–g. The CNF were found to have negligible MA capabilities with a RL_min_ value of only − 11.29 dB and an EAB of 1.34 GHz. The MXene sample displayed a remarkable RL_min_ value of − 29.41 dB. However, its high electrical conductivity caused impedance mismatch, resulting in a limited EAB of only 2.34 GHz. In contrast, the MXene/C aerogels exhibited superior MA performance compared to both CNFs and MXene. Among the samples, MC-1 aerogel demonstrated a RL_min_ = − 40.73 dB at 2.8 mm under the frequency of 8.24 GHz, while achieving an EAB of 5.28 GHz (7.44–12.72 GHz) at 2.70 mm, covering the entire X-band. Similarly, MC-2 aerogel achieved the RL_min_ = − 51.55 dB at 2.3 mm at 9.28 GHz, while obtaining an EAB of 4.72 GHz at 2.4 mm. Among them, MC-3 aerogel exhibited the smallest RL_min_ value with a RL_min_ of − 53.02 dB at 9.28 GHz and a matching thickness of 3.8 mm. To assess the EM capacity accurately, we calculated the ORL_1_ value (RL/filler loading) while considering the filler loading. As shown in Fig. 5g, the MXene/C aerogels exhibit exceptional MA performance in comparison to previously reported MXene-based MA materials [24, 37, 43, 50–53]. They achieved an ORL_1_ value of 343.67 dB and an EAB value of 4.4 GHz. These results were obtained using a filler loading of 15 wt% and a thickness of 2.3 mm.

The electromagnetic parameters can elucidate the MA mechanism of MXene/C aerogels. The real part permittivity (*εʹ*) represents the storage ability of electric energy, while the imaginary part permittivity (*ε″*) represents the dissipation capacity of electric energy [25]. The data in Fig. S6a shows that MC-1, MC-2, and MC-3 aerogels had average *εʹ* values of 8.22, 10.22, and 12.72, respectively. Meanwhile, Fig. S3b shows that the average *ε″* values for MC-1, MC-2, and MC-3 aerogels were 4.29, 4.30, and 6.79, respectively. In comparison, the permittivity of MXene (Fig. S7b) is substantially higher than that of MXene/C aerogel, with real and imaginary parts reaching 42.20 and 12.27, respectively. This difference is primarily attributed to the high electrical conductivity of MXene [11]. The storage (*εʹ*) and dissipation (*ε″*) capabilities of the MXene/C aerogels increased with the MXene content, and therefore, MC-3 exhibited the highest attenuation ability for EM waves. Furthermore, the permittivity of MXene/C aerogels indicated significant fluctuations in the X-band, which may be attributed to the polarization relaxation behavior in this frequency range.

The Cole–Cole curve, derived from the Debye relaxation theory, describes the relationship between the real and imaginary parts of the permittivity as shown in Eq. (4) [16]. It is utilized to determine the polarization relaxation behavior in MXene/C aerogels.

4 $$(\varepsilon^{\prime } - (\varepsilon_{s} + \varepsilon_{\infty } )/2)^{2} + (\varepsilon^{\prime \prime } )^{2} = ((\varepsilon_{s} - \varepsilon_{\infty } )/2)^{2}$$

Here, *ε*_*s*_ is the static permittivity and *ε*_*∞*_ is the relative permittivity at the limiting high frequency. Generally, each Cole–Cole semicircle corresponds to a Debye polarization process. In Fig. 5h, three Cole–Cole semicircles can be observed for MXene/C aerogels, which are possibly attributed to interface polarization relaxation caused by the MXene/C heterojunction and dipole polarization relaxation induced by the abundant functional groups of MXene and carbon. The attenuation coefficient (*α*) is regarded as a crucial parameter influencing the material’s MA performance, and can be expressed by Eq. (5) [54]:

5 $$\alpha = \frac{\sqrt 2 \pi f}{c} \times \sqrt {(\mu^{\prime \prime } \varepsilon^{\prime \prime } - \mu^{\prime } \varepsilon^{\prime } ) + \sqrt {\left( {\mu^{\prime \prime } \varepsilon^{\prime \prime } - \mu^{\prime } \varepsilon^{\prime } } \right)^{2} + (\mu^{\prime } \varepsilon^{\prime \prime } + \mu^{\prime \prime } \varepsilon^{\prime } )^{2} } }$$

The attenuation coefficient *α* curve of the MXene/C aerogels is presented in Fig. 5i. The CM-3 aerogel has a much higher *α* value than MC-1 and MC-2 aerogel, indicating the superior microwave loss capability of MC-3 aerogel. To conclude, increasing MXene content can improve the EM waves attenuation capabilities of the MXene/C aerogel.

**Fig. 6:**
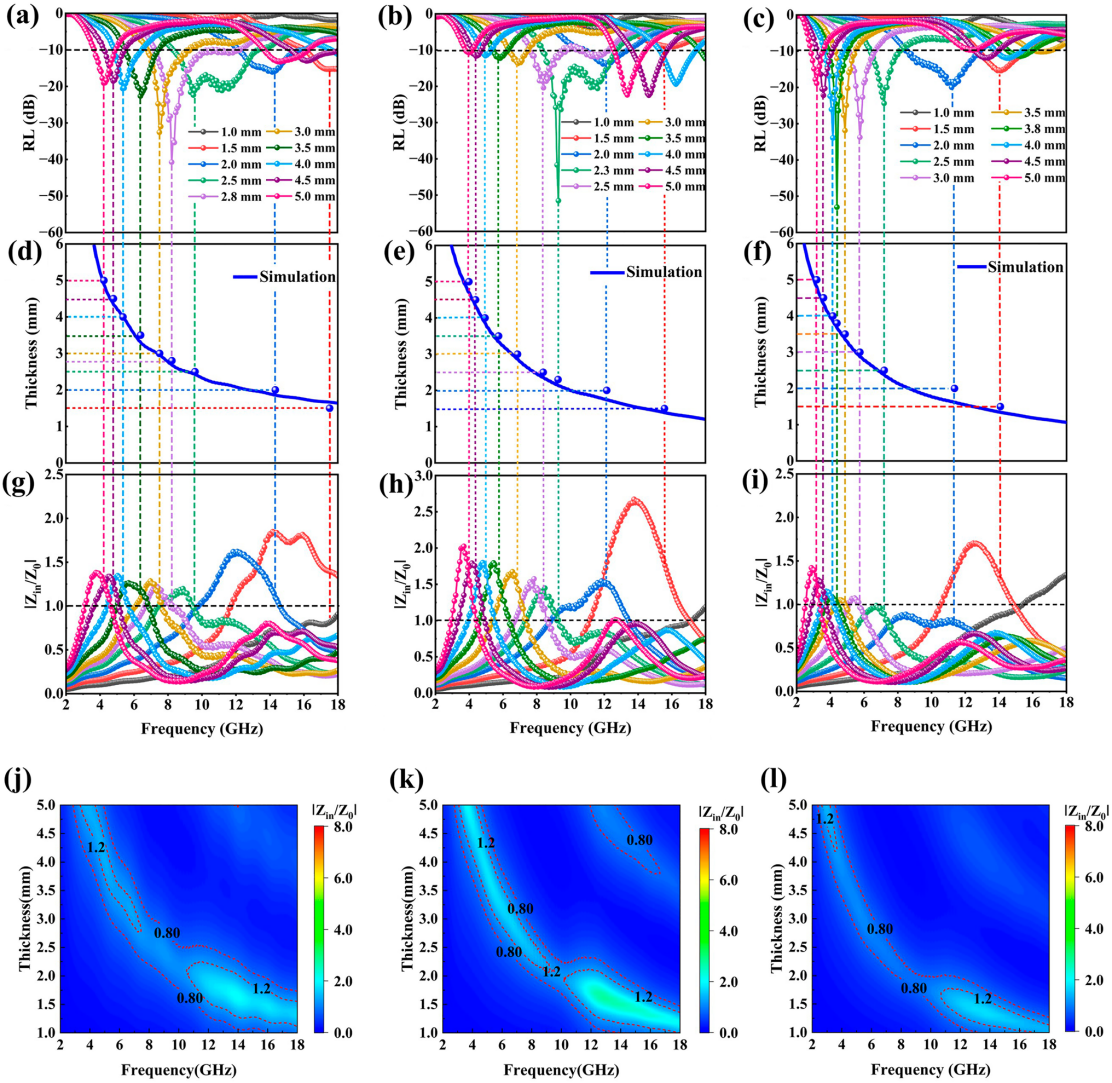
RL-frequency curves: **a** MC-1, **b** MC-2 and **c** MC-3. Relationship between simulation thickness and peak frequency: **d** MC-1, **e** MC-2 and **f** MC-3. Relationship between |*Z*_in_*/Z*_0_| and frequency: **g** MC-1, **h** MC-2 and **i** MC-3. 2D representation of *Z* values of MXene/C aerogels: **j** MC-1, **k** MC-2 and **l** MC-3

For efficient absorption of EM waves, it is crucial for the normalized characteristic impedance value (*Z*) to approach 1, which enables the EM waves to enter the absorbing material without reflection [55]. To understand the mechanism behind the MA performance enhancement of MXene/C aerogels, the *Z* value was calculated using Eqs. (6 and 7) [56], as contained in Fig. 6g–i:

6 $$Z = \left| {Z_{{{\text{in}}}} /Z_{0} } \right|$$

7 $$Z_{{{\text{in}}}} = (\mu_{r} /\varepsilon_{r} )^{1/2} Z_{0}$$

Furthermore, we have depicted the frequency dependence of the RL value of MXene/C aerogels, as shown in Fig. 6a–c. It is noteworthy that nearly all RL_min_ values corresponded to *Z* values close to 1, which allows for efficient absorption of EM waves. The peak value of RL shifted toward lower frequencies as the sample thickness increased, which can be explained by quarter-wavelength attenuation, as described by Eq. (8) [57]:

8 $$t_{m} = \frac{n\lambda }{4} = \frac{nc}{{4f_{m} \sqrt {\left| {\varepsilon_{r} } \right|\left| {\mu_{r} } \right|} }}\quad (n = 1, 3, 5, \ldots )$$

where *f*_*m*_ is the frequency at peak RL, *t*_*m*_ is the sample thickness, *λ* is the EM wavelength, and *n* = 1, 3, 5…. Figure 6d–f demonstrate that the *f*_*m*_ and *t*_*m*_ simulation curve of MXene/C aerogels satisfy the quarter-wavelength matching condition, resulting in RL peaks. For efficient absorbers, their *Z* values typically range from 0.8 to 1.2 [58]. By comparing the 2D *Z*-value distribution plots of MC-1, MC-2, and MC-3 aerogel, Fig. 6j–l, it is evident that MC-1 has a broader frequency band which has the *Z* values between 0.8 and 1.2. This finding provides a compelling reason for the broad EAB (5.28 GHz) observed in MC-1, even though it has a bigger RL_min_ value than other samples. Figure S5f illustrates the normalized characteristic impedance value (*Z*) of MXene, which can reach a maximum of 38.5. However, the maximum *Z*-value of MXene/C aerogels drops to only 3. This significant reduction in the *Z*-value of MXene/C aerogels compared to MXene suggests that this strategy effectively optimizes the impedance matching of MXene.

### Microwave Absorption Mechanism

**Fig. 7:**
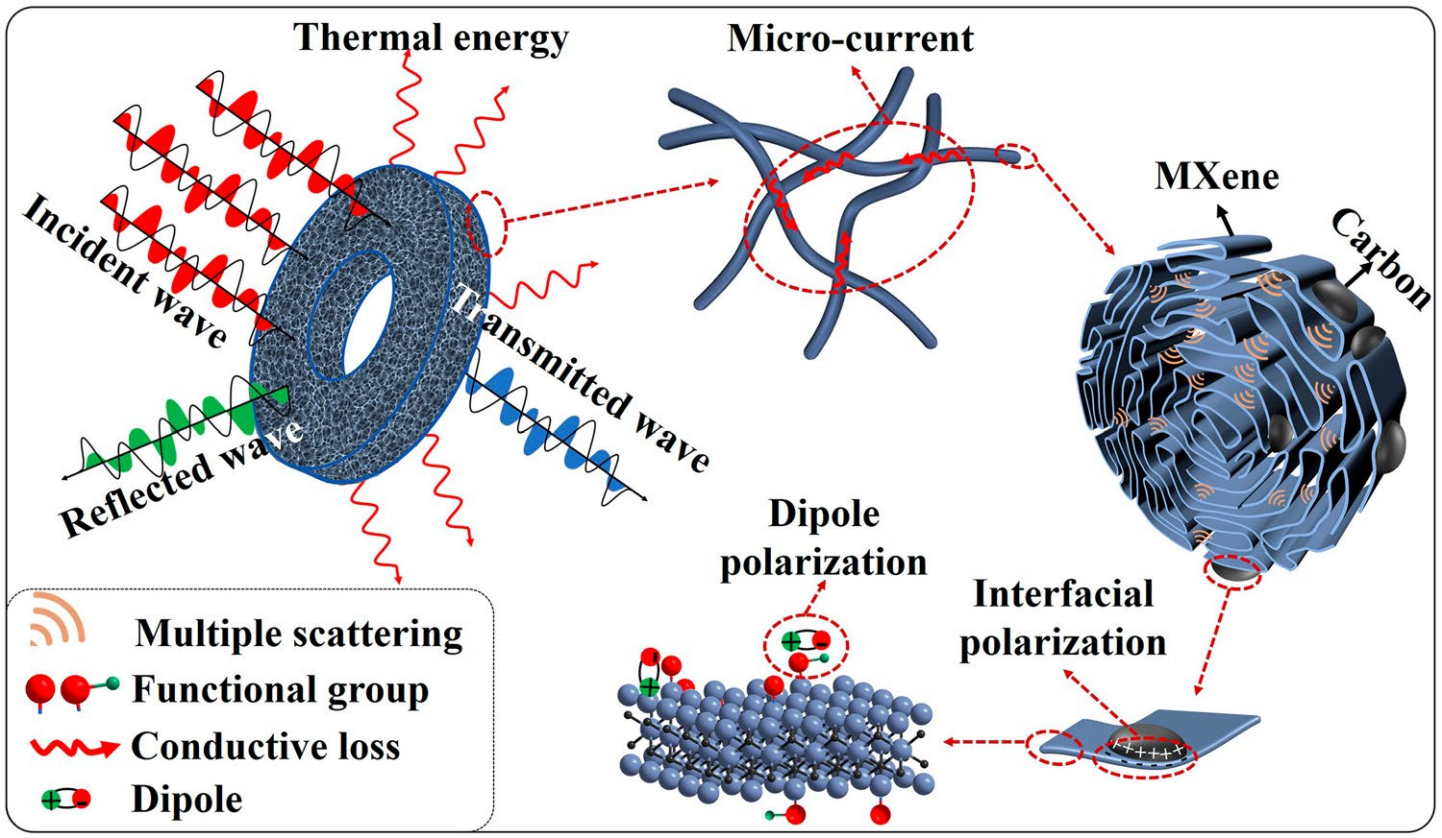
Schematic illustration of MA mechanisms for MXene/C aerogels

Figure 7 presents a possible mechanism for the MA behavior of MXene/C aerogels. The 3D network of MXene/C aerogel creates abundant pores that optimize the material’s impedance matching, and significantly prolongs the microcurrent’s transmission path which leads to stronger conductive loss for EM waves. Additionally, the high porosity 3D network and the embedded MXene nanosheets both serve as sources of multiple reflections and scattering, ultimately leading to efficient EM waves dissipation. By curling MXene nanosheets into 1D nanofibers, the self-stacking of MXene nanosheets is effectively limited. As a result, MXene nanosheets can more uniformly composite with carbon to form a large number of MXene/carbon heterojunctions, which may induce space charge and result in interface polarization. In addition, abundant functional groups of MXene, and the defects in carbon are also beneficial to generate significant dipole polarization, contributing to the effective absorption of EM waves. Therefore, benefiting from its unique structural advantages and the synergistic effect of its components, the MXene/C aerogel displays remarkable MA performance.

### RCS Simulation Results

**Fig. 8:**
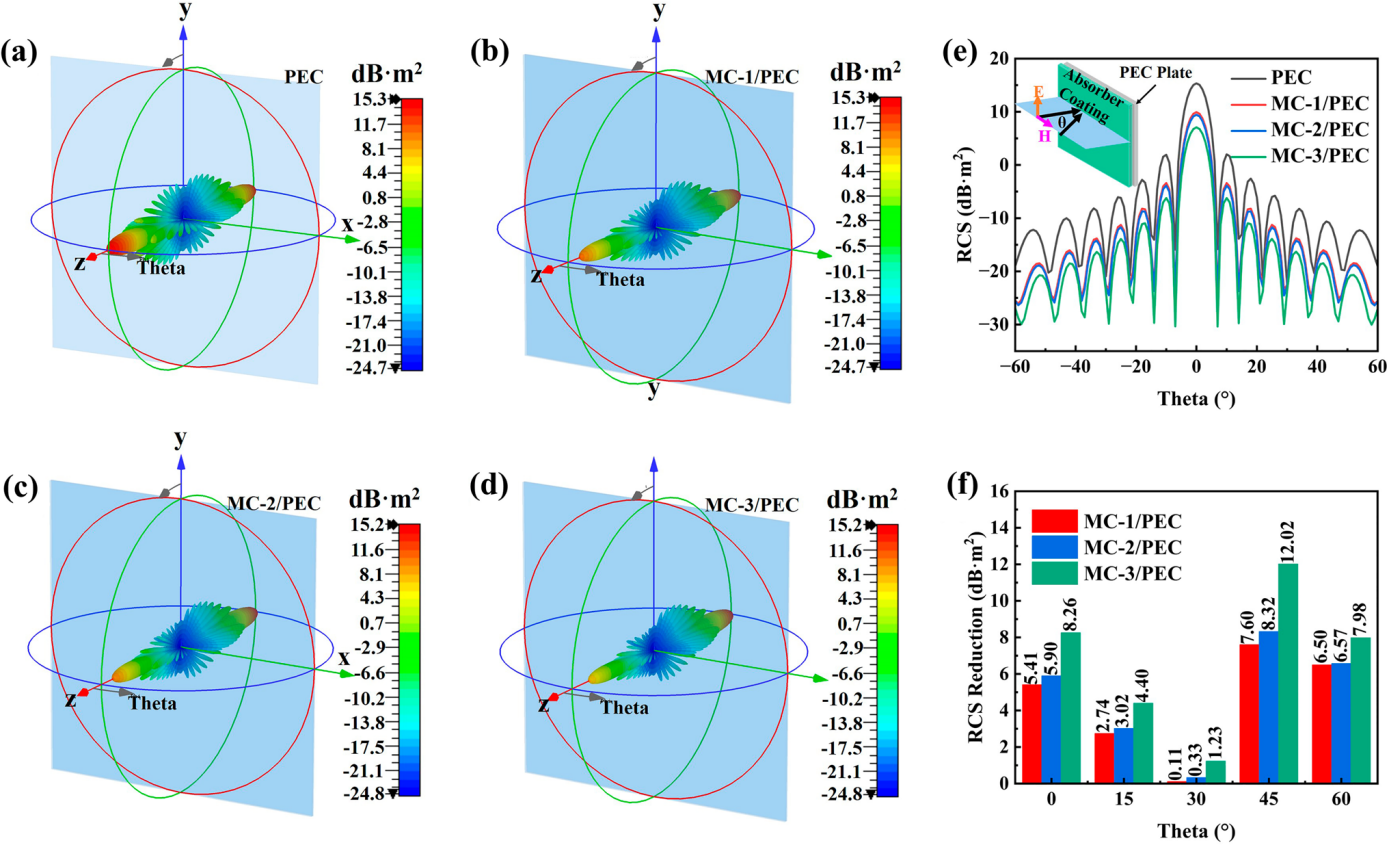
CST simulation results of **a** PEC, **b** MC-1/PEC, **c** MC-2/PEC, and **d** MC-3/PEC. **e** Simulated RCS curves of the PEC and MXene/C aerogels at the scattering angle of − 60° to 60°. **f** RCS reduction values of MC-1/PEC, MC-2/PEC and MC-3/PEC

To evaluate the MA capacity of MXene/C aerogels under real far-field conditions, the RCS of PEC plates coated with MC-1, MC-2, and MC-3 aerogel were simulated using CST software. As shown in Fig. 8a–d, the PEC plate displayed the highest radar scattering intensity, while the PEC plate covered with MXene/C aerogels demonstrated a significant reduction in radar scattering intensity, particularly for the MC-3/PEC with the lowest RCS value. Figure 8e illustrates the RCS curves of a PEC plate and PEC plates coated with aerogel, with scattering angles ranging from − 60° to 60°. The RCS values exhibit a noticeable decrease from MC-1/PEC to MC-3/PEC, as evidenced by the curves, which is consistent with the MA performance of the samples presented in Fig. 5. In addition, Fig. 8f shows the RCS reduction values of PEC plates coated with aerogel compared to uncoated PEC plates at different incident angles. The results demonstrate that MXene/C aerogels are effective in absorbing radar waves, thereby achieving a superior radar stealth effect. The MC-3/PEC model exhibit the maximum RCS reduction value of 12.02 dB m^2^ at 45°.

### Thermal Insulation Performance

**Fig. 9:**
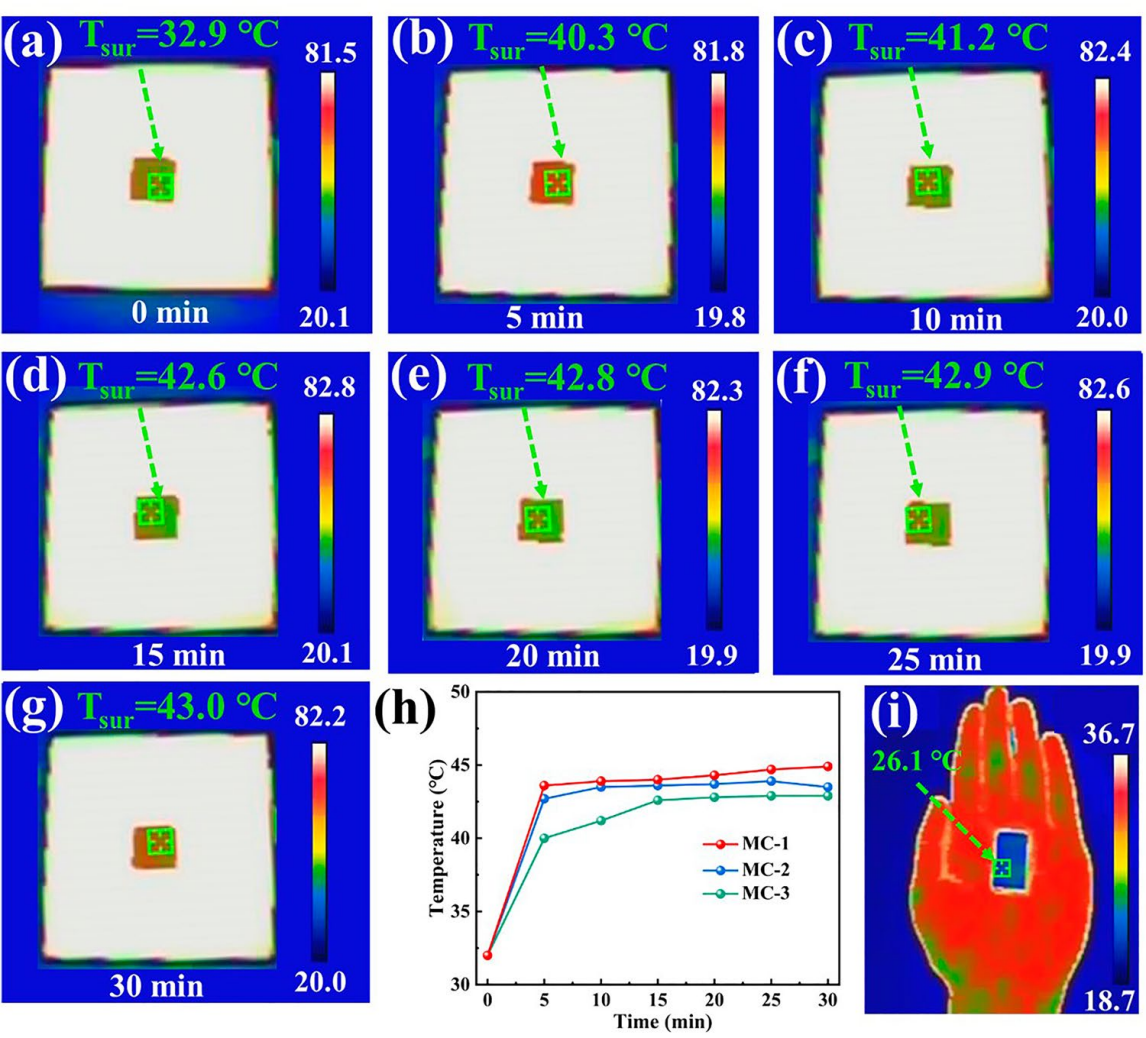
**a**–**g** Thermal infrared images of MC-3 captured at intervals of 5 min from 0 to 30 min. **h** Surface temperature change curves of the aerogels on the hot plate of 82 °C. **i** Thermal infrared image of MC-3 placed on the hand

MXene/C aerogels have the ability to trap air, resulting in exceptional thermal insulation. To evaluate their thermal insulation capability, aerogels with a 5-mm thickness were placed on a flat hot plate set to 82 °C, and an infrared thermometer was used to measure their surface temperatures. As an example, in Fig. 9a–k, the infrared images of the MC-3 during continuous heating for 0–30 min demonstrate a gradual increase in surface temperature. After 30 min, the temperature differences between the aerogels’ surfaces and the hot plate of aerogels were all greater than 30 °C, as shown in Fig. 9h, demonstrating excellent thermal insulation performance. Furthermore, when the aerogel is placed on the hand, the thermal infrared image (Fig. 9i) reveals that the upper surface of the aerogel appears dark color and seamlessly blends with the surrounding environment. This observation highlights the exceptional thermal infrared stealth capability of the MXene/C aerogels.

## Conclusion

We have developed a novel strategy to curl the 2D MXene nanosheets into 1D nanofibers using electrospinning, and subsequently connect the nanofibers to fabricate MXene/C aerogels with a 3D conductive network. This 3D network can optimize the impedance matching and EM waves loss capacity, resulting in exceptional MA performance. The MXene/C aerogels achieved the RL_min_ of − 53.02 dB (*f* = 4.44 GHz, *t* = 3.8 mm) and EAB of 5.3 GHz (*t* = 2.4 mm, 7.44–12.72 GHz). The RCS simulation results show that the RCS reduction value of MXene/C aerogels can reach 12.02 dB m^2^. In addition, the 3D network renders the MXene/C aerogels with remarkable thermal insulation and infrared stealth capabilities. Consequently, the lightweight and multifunctional MXene/C aerogels developed in this work show great potential for MA materials and other applications, such as thermal insulation and infrared stealth.

### Supplementary Information

Below is the link to the electronic supplementary material.

Supplementary file1 (PDF 1046 KB)
