# Supplementary material for: Multifunctional MXene/C Aerogels for Enhanced Microwave Absorption and Thermal Insulation
Source: Nanomicro Lett. 2023 Aug 9;15:194. doi: 10.1007/s40820-023-01158-7 (PMC10412520; doi:10.1007/s40820-023-01158-7)
Supplement: Supplementary file 1 — Supplementary file1 (PDF 1046 KB) [file 40820_2023_1158_MOESM1_ESM.pdf]

Supporting Information for

Multifunctional MXene/C Aerogels for Enhanced Microwave Absorption and Thermal Insulation

Fushuo Wu[1], Peiying Hu[1], Feiyue Hu[1], Zhihua Tian[1], Jingwen Tang[1], Peigen Zhang[1, *], Long Pan[1], Michel W. Barsoum[2], Longzhu Cai[3], ZhengMing Sun[1, *]

[1] School of Materials Science and Engineering, Southeast University, Nanjing 211189, P. R. China

[2] Department of Materials Science & Engineering, Drexel University, Philadelphia, PA 19104, USA

[3] The State Key Laboratory of Millimeter Waves, School of Information Science and Engineering, Southeast University, Nanjing 210096, P. R. China

*Corresponding authors. E-mail: zhpeigen@seu.edu.cn (Peigen Zhang); zmsun@seu.edu.cn (ZhengMing Sun)

Supplementary Figures

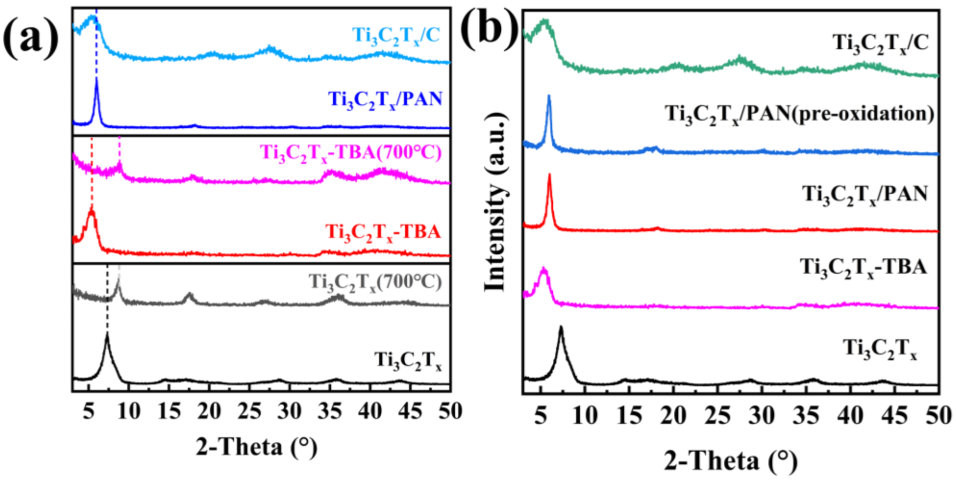

Fig. S1 a XRD patterns of $Ti_3C_2T_x$, $Ti_3C_2T_x$-TBA and $Ti_3C_2T_x$/PAN before and after high temperature treatment at 700 ℃. **b** XRD patterns of $Ti_3C_2T_x$, $Ti_3C_2T_x$-TBA, $Ti_3C_2T_x$/PAN, $Ti_3C_2T_x$/PAN (pre-oxidation) and $Ti_3C_2T_x$/C

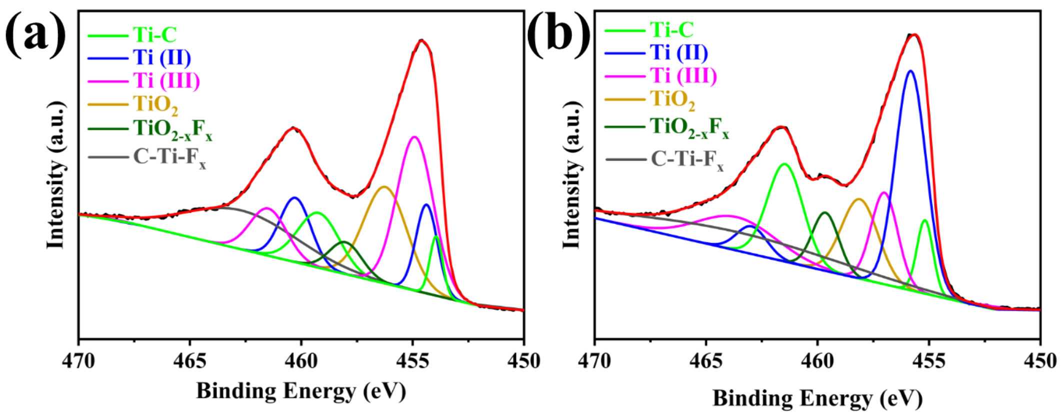

Fig. S2 Ti 2p XPS spectrum of **a** MXene and **b** MC-1

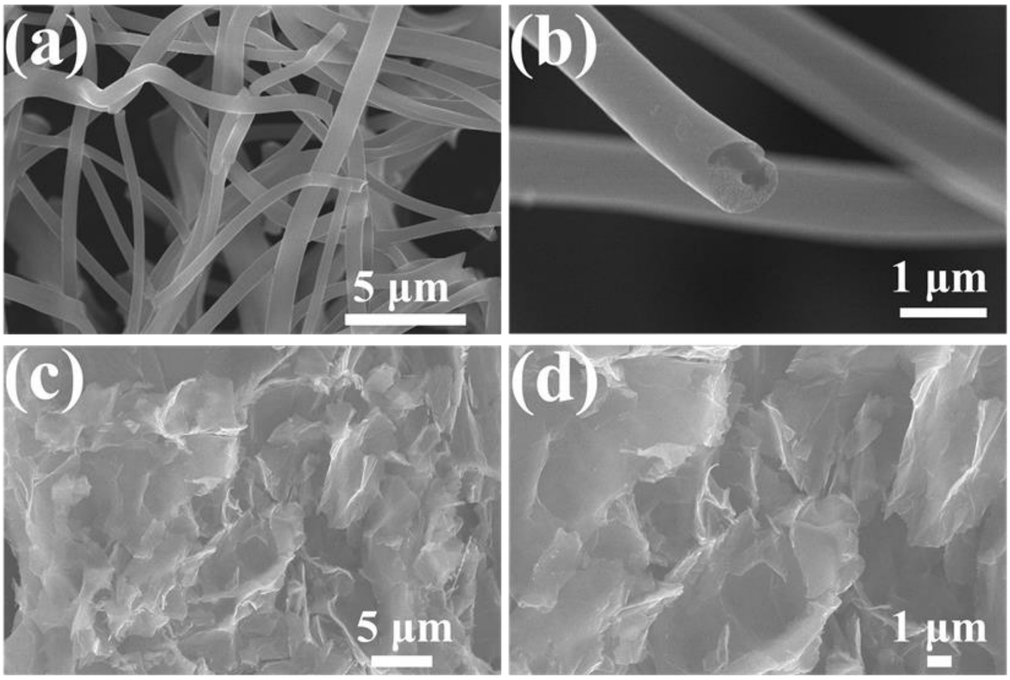

Fig. S3 SEM images of the **a, b** carbon nanofiber and **c, d** $Ti_3C_2T_x$ MXene nanosheets

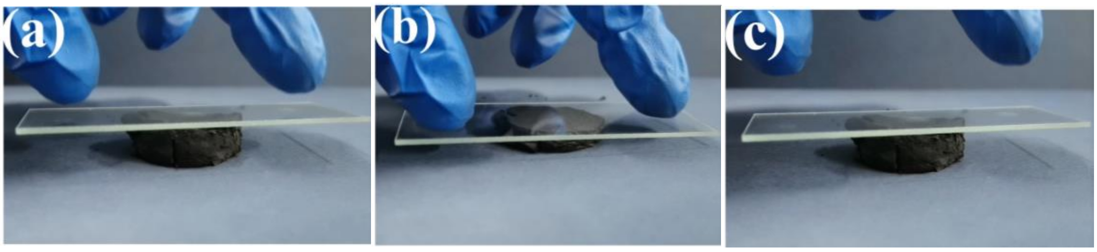

Fig. S4 Digital images of the compression process of MXene/C aerogels: **a** initial state, **b** compressed state, **c** recovered state

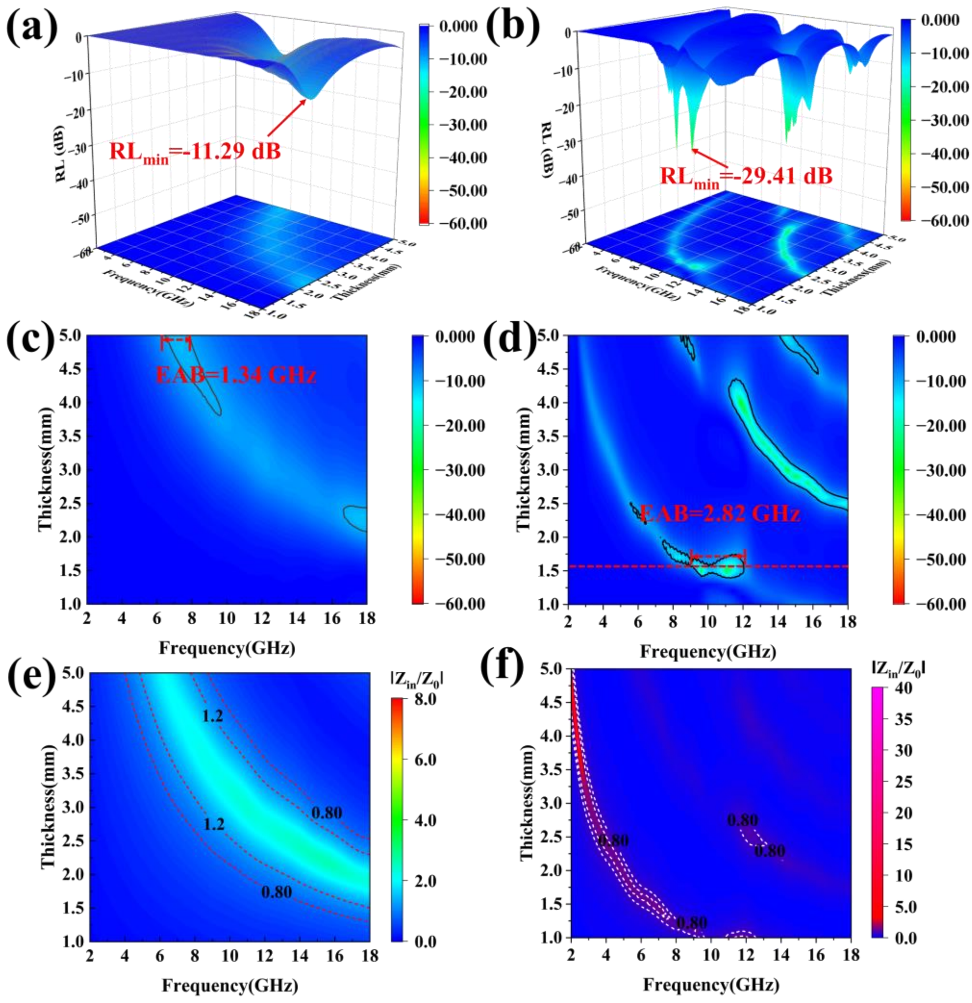

Fig. S5 3D representation of RL of **a** Carbon nanofiber and **b** MXene. 2D representation of RL values of **c** Carbon nanofiber and **d** MXene. 2D representation of Z values of **e** CNF and **f** MXene

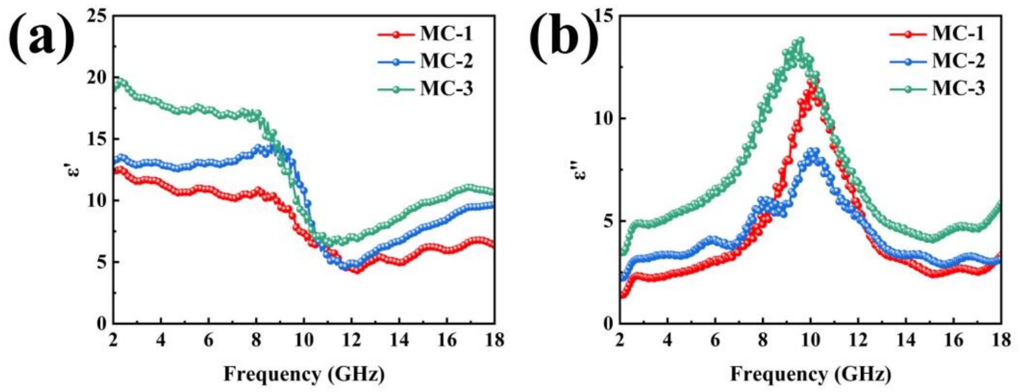

Fig S6 The real part **a** and imaginary part **b** of permittivity for MXene/C aerogels

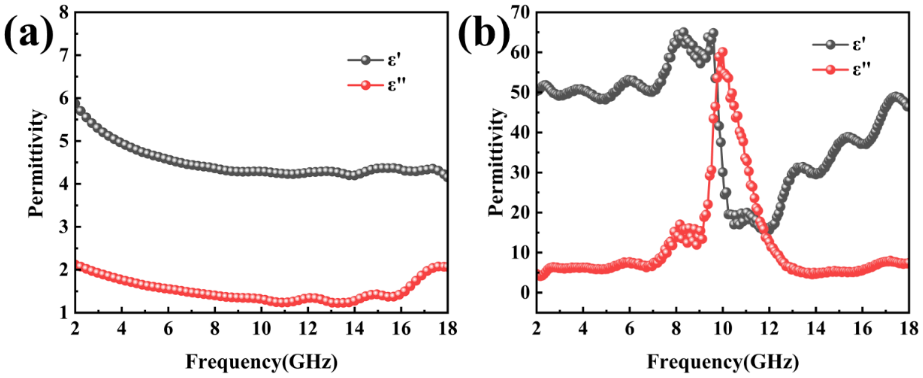

Fig. S7 The permittivity of **a** CNF and **b** MXene
